# Extracellular matrix metalloproteinase expression in endometrial tissue after arterial embolization of myomas

**DOI:** 10.6061/clinics/2021/e2145

**Published:** 2021-01-18

**Authors:** Franco Loeb Chazan, Tatiana C.S. Bonetti, Mariano Tamura Vieira Gomes, Vinicius Adami Vayego Fornazari, Manoel João Batista Castello Girão, Claudio Emilio Bonduki

**Affiliations:** I Departamento de Ginecologia, Universidade Federal de Sao Paulo, Sao Paulo, SP, BR; II Departamento de Diagnostico por Imagem, Universidade Federal de Sao Paulo, Sao Paulo, SP, BR

**Keywords:** Leiomyoma, Extracellular Matrix, Matrix Metalloproteinase, Arterial Embolization of Myomas

## Abstract

**OBJECTIVES:**

Arterial embolization of myomas (AEM) is controversial because of the changes that occur in the extracellular matrix (ECM) of the endometrium and its effect on gestational success in infertile patients desiring reproductive capability. Therefore, we performed this study on the expression of genes in the ECM of the endometrium, such as those coding metalloproteinases (MMP), before and 6 months after embolization of the uterine arteries.

**METHODS:**

Seven women with leiomyomas were evaluated, and MMP3 and MMP10 levels were measured. The women underwent pelvic nuclear magnetic resonance (NMR), examination, and endometrial biopsy between the 20^th^ and 24^th^ day of the menstrual cycle, and pre- and post-AEM (after 6 months). For data analysis, the Cq comparative method, also known as the 2-ΔΔCT method, was used to calculate the relative quantities of *MMP* gene expression among the samples collected.

**RESULTS:**

There was a significant decrease by 9.52 times in the expression of *MMP3* (*p*=0.007), and a non-significant change in the expression of *MMP10* (*p*=0.22) in post-AEM-treated women than pre-AEM-treated women.

**CONCLUSIONS:**

The results suggest that ECM continues to undergo tissue remodeling 6 months after AEM, at least with regard to *MMP3* expression, suggesting that AEM affects the ECM for at least 6 months after the procedure.

## INTRODUCTION

Uterine leiomyomas are benign smooth muscle tumors that develop from myometrial cells by an increase in extracellular matrix (ECM) components such as fibronectin, proteoglycans, and collagen. It is the most frequent pelvic tumor, and its incidence (5-80%) depends on the population studied and the diagnostic method used. A study using transvaginal ultrasound in a population of women aged 25-40 years found an incidence of 3.3% among women aged 25-32 years and 7.8% between 33-40 years, demonstrating the influence of age on the prevalence of uterine leiomyoma (1). At approximately 50 years of age, there is a cumulative incidence of 70-80% (2,3). However, the high incidence in some studies may be related to the selected population, especially patients over 40 years old, although many are asymptomatic (4,5). According to the Ministry of Health's Computerized Hospitalization System, 107,293 hysterectomies were performed in Brazil between November 2016 and December 2017, of which 58.2% were due to uterine leiomyomas (6).

The hormones estrogen and progesterone seem to be the main promoters for the development of leiomyoma. These hormones act directly on receptors and indirectly affect the expression of growth hormones and apoptosis factors. Ultrasonography is the diagnostic method of choice because of its high accuracy, high sensitivity (95-100%), and low cost (5). Hysteroscopy, hysterosonography, hysterosalpingography, computed tomography, and nuclear magnetic resonance (NMR) can be used as complementary techniques. However, these methods are limited to specific cases, for example, when it is not possible to delimit large-volume nodules. In these cases, NMR is more accurate for pre-surgical evaluation (5).

Arterial embolization of myomas (AEM) has been used since the late 1970s, with the first publication on treatment of uterine leiomyoma published in 1995 (7). On average, an improvement of 80-90% of symptoms is seen and the volume of myomas is reduced by 40-50%; however, recurrences are not rare (8). A systematic review comparing AEM, hysterectomy, and myomectomy using meta-analysis and seven randomized trials showed that patients who underwent AEM had shorter hospital stays and faster return to activities (1).

ECM metalloproteinases (MMP) are involved in matrix rupture during physiological processes such as embryonic development, reproduction, and tissue remodeling. Degradation and remodeling of the ECM are also key events in human reproduction (9,10). Hilario et al. (11) evaluated gene expression related to endometrial receptivity in patients undergoing AEM, concluding that there was under-expression of the *LIF*, *IL-11*, and *Hoxa-11* genes, and that the expression of IL-6, claudin-4, Hoxa-10, estrogen, and progesterone receptors in the endometrium did not change significantly after AEM. Bernardo et al. (10) screened 86 ECM genes and cytokines and found overexpression of some MMPs after AEM. Thus, this study showed MMP changes after AEM. Therefore, eventual changes in endometrial flow in women who have undergone AEM can alter ECM gene expression, promoting changes in tissue reproductive function and affecting fertility. On the other hand, the post-procedural improvement in symptoms, diagnosis, and therapeutic resources boosted the technique and created positive expectations regarding female reproductive capacity. With regard to maintenance of reproductive capacity after AEM, several studies have shown pregnancy rates around 30% after this procedure (12-14). However, it would be interesting to know the timing of ECM remodeling after AEM by studying MMP expression.

Therefore, this work aimed to analyze endometrial gene expression of *MMP3* and *MMP10* in patients with reproductive desire before and six months after AEM since the mechanism of interaction between the embryo and the endometrium is multifactorial, complex, and still under debate.

## METHODS

### Patients

Seven women, who met the inclusion criteria of the research protocol, were selected at the uterine myoma outpatient clinic of the Gynecology Department of the Escola Paulista de Medicina of the Federal University of São Paulo (EPM-UNIFESP). The analyzed patients’ mean age was 32.29 years (25-38 years), with an average uterine volume of 355.9 cc^3^ (155 cc^3^-874 cc^3^; Table 1).

The study was approved by the Research Ethics Committee of the Federal University of São Paulo (CEP-UNIFESP) (CEP-1246/2011) and, after clarification of all available therapeutic options and establishment of a consensus between the medical team and patient, the informed consent form was signed.

### Inclusion and exclusion criteria

Inclusion criteria for participation in the study were: abnormal uterine bleeding, dysmenorrhea, pelvic pain, urinary discomfort, deep dyspareunia, and a desire to maintain reproductive capacity with no possibility for other conservative treatment.

Exclusion criteria were genital neoplasia, use of hormonal contraceptive method, use of hormones to regulate menstrual cycle, pregnancy, acute pelvic inflammatory disease, coagulopathy or vascular disorders, prior pelvic irradiation, nephropathy, and liver diseases.

### Magnetic resonance imaging of the pelvis (MRI), with intravenous contrast

The women underwent an MRI for proper assessment of the myoma nodules in terms of size, number, and location, and to perform the differential diagnosis of adenomyosis (15).

### Endometrial biopsy

Endometrial biopsy was performed with a modified Novak curette during the second menstrual phase (secretory phase), between the 20^th^ and 24^th^ days of the menstrual cycle, based on the average number of days of previous menstrual cycles for each patient. Sample collection was performed at the gynecology clinic, without the use of analgesia.

Each patient and their respective sample were identified with the numbers 1 to 7. The collected material was stored at -80°C until further ribonucleic acid (RNA) extraction.

### Arterial embolization of myomas

The AEM procedure was performed using spherical and non-spherical gelatinous micro-particles (biospheres) with diameters ranging from 500 μm to 900 μm. The procedure cut-off point was when the contrast injection flow in the uterine arteries decreased, characterized by the “end point” image. Special attention was given to the possibility of embolization of ovarian arteries, in which the prevalent blood flow sometimes originates in the uterine arteries. 

After a day or two, the patients were discharged.

### Reevaluation post-AEM

A reevaluation of the patients was scheduled 7 days after AEM for clinical post-procedural assessment and general guidance. A new assessment was scheduled 6 months after the AEM and consisted of anamnesis, physical examination, MRI examination, and endometrial biopsy.

### Extraction and isolation of RNA in real-time

RNA extraction was performed using the TRIzol method for cryopreserved tissue. Before thawing, the macerate was transferred to a microtube containing 1 mL of a single-phase solution of phenol and guanidine isothiocyanate (TRIzol). The macerate was placed at room temperature for 15 min and in a vortex for 15 min, as per the manufacturer's protocol. Chloroform (200 µL) was added to the sample, which was agitated for 30 min, placed at room temperature for 3 min, and then centrifuged at 12,000 rpm for 15 min at 4°C. The aqueous upper phase was transferred to new microtubes, and 500 µL of isopropanol and 10 µL of 3M sodium acetate were added. After being homogenized by inversion, the microtubes were kept in a -20°C freezer overnight for precipitation. The material was centrifuged at 14,000 rpm for 15 min at 4°C. The precipitate was washed twice with 80% ethanol, vortexed for 2 min at 14,000 rpm and dried for 20 min. The RNA was then dissolved in 50 µL of RNAse-free water and stored at -80°C for future analysis. The quantity and quality of the extracted RNAs were evaluated by spectrophotometry, using Nanodrop equipment (Nanodrop Technologies Inc., Rockland, DE), and expressed in ng/µL.

### Synthesis and quantification of complementary deoxyribonucleic acid (cDNA)

cDNA was synthesized from the extracted RNA using the SuperScript TM VILO TM cDNA Synthesis kit (Invitrogen, Thermo Fisher). The cDNAs obtained from all paired samples were stored at -20°C until real-time polymerase chain reaction (PCR) was performed.

### Real-time PCR

Paired samples were evaluated for gene expression by real-time PCR using the TaqMan^®^ Gene Expression Assay (Applied Biosystems), with the primers described in the literature for the *MMP3* (Hs00968305_m1) and *MMP10* (Hs00233987_m1) genes. The genes *BACTH* (Hs99999903_m1) and *GAPDH* (Hs 02786624_g1) were used as normalizers of the reaction because they are endogenous genes.

Total mRNA (100 ng) was used for each PCR reaction in a total volume of 25 µL as per the manufacturer's protocol. The cycling conditions were as follows: 30s at 48°C, 10 min at 95°C; and 50 cycles of 15 s denaturation at 95°C and 60s of annealing at 60°C. The values of the quantification cycles (Cq), defined as the number of PCR cycles in which the fluorescence generated by amplification crossed the threshold, were used as an end point. Three repetitions per sample were performed to ensure statistical significance. For data analysis, the fold change (FC) comparative method, FC=2-ΔΔCT, was used to calculate the relative quantities of gene expression between samples**.**

### Statistical analysis

The average of the experimental triplicates was used to normalize the expression. FC=2-ΔΔCT of the pre- and post-embolization samples were compared using the paired student’s t-test. Values of *p*<0.05 were considered statistically significant. The values of relative expression between the two groups were calculated using the comparative method (Relative expression=2ˆ(-deltadeltaCt); where deltadeltaCt=mean deltaCt post-AEM-mean delta Ct pre-AEM).

## RESULTS

The demographic data of the patients analyzed in relation to age, ethnicity, and uterine volume are shown in Table 1. The analyzed patients were 32.29+1.91 years old, with a uterine volume of 355.9+90.04 cc^3^, and of varied ethnicity (four Africans, two Asians, and one Caucasian).

**Figure 1:**
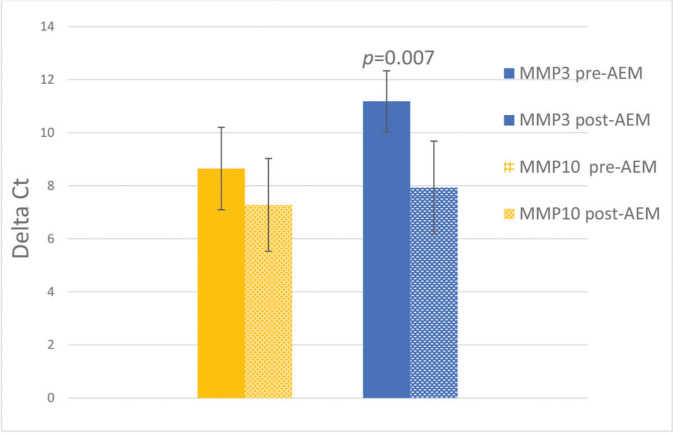
Relative expression of *MMP3* and *MMP10* before and after arterial embolization of myomas (AEM) (mean±SEM); N=7. The Y-axis represents the deltaCt values (mean Ct of the target gene-mean Ct of the endogenous genes). The vertical lines indicate the mean and standard error for each of the samples. *p=0.007.

The mean values of *MMP3* and *MMP10* expression, before and after AEM, and the standard errors are shown in Figure 1, with *p*<0.05 indicating statistical significance. There was a decrease in *MMP3* expression from 11.18±1.15 to 7.93±1.09 (*p*<0.05) and a non-significant change from 8.65±1.55 to 7.28±1.75 for *MMP10* (*p*>0.05). The difference in expression (relative expression/FC) of *MMP3* observed pre- and post-AEM was 9.52 times (Figure 1).

**Figure 2:**
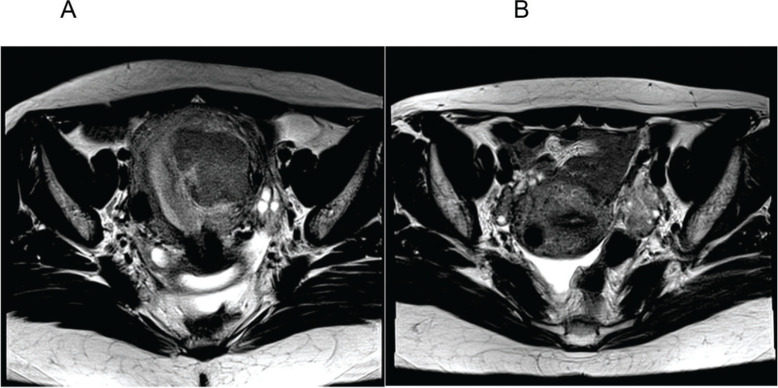
A. Axial section of the pelvis before arterial embolization of myomas (AEM) showing a submucosal mass with an intramural component; B. Axial section of the pelvis 6 months after AEM showing absence of the previously characterized myometrial mass and a subserous residual fundic/cicatricial nodule.

Figure 2 represents the pre- (Figure 2A) and post-AEM (Figure 2B) findings in one patient, revealing the removal of the endometrial mass and the residual subserosa.

## DISCUSSION

In this study, we selected a group of women with leiomyoma, with uniformity in terms of symptoms and age, to analyze the expression of MMP pre- and post-AEM. We observed under-expression of *MMP3*, but not *MMP10,* in the ECM. We also noticed the uniform action of AEM treatment, reflected by similar volumetric decrease in leiomyomas among all patients (mean reduction rate of 36.6%). Another important characteristic was the involvement of nulliparous patients with no history of pregnancy, which ruled out the presence of patient characteristics such as primary infertility that could influence the study results.

The metalloproteinase genes are related to the facilitation of embryonic implantation (7,8,10,16). In our study, we observed an under-expression of *MMP3* 6 months post-AEM. As mentioned above, MMPs favor endometrial reorganization and cell proliferation. Much of the molecular endometrial processes occur through the interaction of the ECM components and metalloproteinases (MMP) during the menstrual cycle, especially during the luteal phase. Therefore, the possibility of infertility after AEM is a matter of debate (17).

AEM has been associated with an increase in adverse obstetric outcomes in a few studies. A meta-analysis study (4) of 227 pregnancies after AEM in nine main observational studies, including a randomized trial comparing pregnancies in groups matched for patient age and myoma location, revealed that pregnancy after AEM was associated with a significant increase in abortion rates (35% *versus* 17%). Therefore, given our results, we assume that ECM continues to undergo tissue remodeling, mainly for MMP3 and not for MMP10. Embryo implantation, invasion, and placentation are complex molecular processes that involve several other molecules, and enzymatic interactions have not been completely elucidated until now. Further studies should be carried out to confirm the adverse effects of AEM on pregnancy.

### Study limitations

The limitations in this study were the difficulties in working with extremely sensitive material (RNA/cDNA), and the quality and quantity of the biopsy material, as they were blindly (not guided by hysteroscopy) obtained without analgesia. The stratification of the study population in terms of age, race and parity, and the small sample size also limited the study. For further studies, endometrial biopsy should be performed using hysteroscopy. It is important to follow-up on patients undergoing AEM and their pregnancy rates, as AEM is performed only in patients with reproductive desire.

## AUTHOR CONTRIBUTIONS

Chazan FL performed the design, preparation, data collection, transcription, and manuscript organization. Bonetti TCS performed molecular analysis of the samples. Gomes MTV and Girão MJBC reviewed the manuscript. Fornazari VAV performed image processing of the data. Bonduki CE performed the design of the experiments and manuscript review.

## Figures and Tables

**Table 1:** Demographic data of the patients analyzed (N=7).

Patients	Characteristics
Age (years)	32.29±1.91
Uterine Volume (cc^3^)	355.9±90.04

Mean±SEM.
